# Enhanced Release Probability without Changes in Synaptic Delay during Analogue–Digital Facilitation

**DOI:** 10.3390/cells13070573

**Published:** 2024-03-26

**Authors:** Sami Boudkkazi, Dominique Debanne

**Affiliations:** 1 Physiology Institute, University of Freiburg, 79104 Freiburg, Germany; 2 Unité de Neurobiologie des Canaux Ioniques et de la Synapse (UNIS), Institut National de la Santé et de la Recherche Médicale (INSERM), Aix-Marseille University, 13015 Marseille, France

**Keywords:** neuronal timing, synaptic transmission, synaptic latency, context-dependent facilitation, neocortex, local circuits

## Abstract

Neuronal timing with millisecond precision is critical for many brain functions such as sensory perception, learning and memory formation. At the level of the chemical synapse, the synaptic delay is determined by the presynaptic release probability (*Pr*) and the waveform of the presynaptic action potential (AP). For instance, paired-pulse facilitation or presynaptic long-term potentiation are associated with reductions in the synaptic delay, whereas paired-pulse depression or presynaptic long-term depression are associated with an increased synaptic delay. Parallelly, the AP broadening that results from the inactivation of voltage gated potassium (Kv) channels responsible for the repolarization phase of the AP delays the synaptic response, and the inactivation of sodium (Nav) channels by voltage reduces the synaptic latency. However, whether synaptic delay is modulated during depolarization-induced analogue–digital facilitation (d-ADF), a form of context-dependent synaptic facilitation induced by prolonged depolarization of the presynaptic neuron and mediated by the voltage-inactivation of presynaptic Kv1 channels, remains unclear. We show here that despite *Pr* being elevated during d-ADF at pyramidal L5-L5 cell synapses, the synaptic delay is surprisingly unchanged. This finding suggests that both *Pr*- and AP-dependent changes in synaptic delay compensate for each other during d-ADF. We conclude that, in contrast to other short- or long-term modulations of presynaptic release, synaptic timing is not affected during d-ADF because of the opposite interaction of *Pr*- and AP-dependent modulations of synaptic delay.

## 1. Introduction

Precise neuronal timing plays a critical role in many brain functions including sensory processing [1,2,3,4,5,6] or the encoding of memory by synaptic plasticity [7,8,9]. In fact, neural information is not solely transmitted in the brain through the modulation of the firing rate (i.e., rate coding), but also transmitted by the fine temporal organization of neuronal discharge (i.e., time coding) [10,11,12,13]. Many experimental and theoretical findings argue for the increasing importance of time coding in the neural function. For instance, sensory information has been found to be adequately encoded by the first action potential latency in the visual, auditory, olfactory and somatosensory systems [14,15,16,17,18,19]. In addition, theoretical works show that time coding based on the first action potential timing is powerful and highly discriminative over other classical forms of neural coding [11,20]. 

In simple neuronal networks, the timing between connected neurons is usually described by the synaptic latency which is the sum of (i) the conduction time of the action potential along the axon that depends on both the axon diameter and the presence of myelin and (ii) the synaptic delay [21]. While the conduction time is usually stable over neural stimulations, synaptic delay is not fixed [22,23]. Rather, it is determined by the calcium concentration in the presynaptic bouton [24,25] and the presynaptic release probability (*Pr*) [26]. The synaptic delay is long for a low calcium concentration or for a low *Pr* whereas it is short for a high calcium concentration or for a high *Pr*. In addition, synaptic delay is modulated by ~0.5–1 ms during short-term plasticity such as paired-pulse facilitation (PPF) and depression (PPD) or during long-term synaptic enhancement (LTP) or depression (LTD) due to changes in *Pr* [26]. This plasticity of synaptic timing is theoretically important for learning and memory [27,28]. Furthermore, synaptic delay is augmented by ~0.5 ms in neocortical neurons following 4-8 days of sensory deprivation [29], confirming that changes in synaptic delay occur following homeostatic adaptation to external modifications and, thus, could constitute a putative code for neural information [26]. 

In parallel, the duration and the amplitude of the presynaptic waveform (i.e., the action potential (AP)) strongly determine the synaptic latency. The calcium influx is maximal during the repolarization phase of the AP [30,31]. Thus, the broadening of the presynaptic spike by blocking potassium channels responsible for the repolarization of the AP prolongs the synaptic delay at cortical and hippocampal synapses, and the reduction in the amplitude of the presynaptic AP shortens the synaptic delay [32]. 

The context-dependent modulation of spike-evoked synaptic transmission (reviews in references [33,34]) has been identified at hippocampal [35,36,37,38,39,40], neocortical [39,40,41,42], auditory [43] and cerebellar synapses [44,45,46,47]. It involves either the voltage inactivation of presynaptic voltage-gated potassium channels [38,42,48], activation of presynaptic voltage-gated calcium channels [45], recovery of axonal Nav from inactivation [39] or minimization of axonal Nav channel inactivation [40]. This form of analogue–digital facilitation is also possible because voltage changes induced in the cell body are electrotonically transmitted in the presynaptic axon to the terminals with an attenuation described by a space constant that depends on morphological characteristics such as the axon diameter and the presence of myelin [34,49]. Slow wave sleep is associated with “down” to “up” transitions of membrane potential that facilitate synaptic transmission [37] due to the inactivation of voltage-gated potassium channel type 1 (Kv1) [38]. In fact, the voltage inactivation of Kv1 channels during depolarization-induced analogue–digital facilitation (d-ADF) broadens the presynaptic AP, increases the spike-evoked calcium influx and, thus, enhances synaptic transmission [41,42,48,50]. Then, it is plausible that the *reduced* synaptic delay due to the elevation in *Pr* would be masked by the *prolonged* synaptic delay caused by the Kv1-dependent broadening of the presynaptic AP.

We show here that despite the increased synaptic strength due to the elevation of *Pr* during d-ADF, the synaptic delay at L5-L5 cortical synapse is surprisingly unchanged on average. As d-ADF is due to a Kv1-dependent broadening in AP width, we conclude that *Pr*- and AP duration-dependent changes in synaptic delay compensate for each other. Thus, in contrast with other short-term or long-term presynaptic dynamics, synaptic delay is not changed during context-dependent facilitation at unitary synapses formed by pairs of L5-L5 neurons.

## 2. Materials and Methods

Cortical slices (350–400 µm thick) were obtained from 13- to 20-day-old Wistar rats of both sexes as previously reported [26]. All experiments were carried out according to the European and Institutional guidelines for the care and use of laboratory animals (Council Directive 86/609/EEC and French National Research Council) and approved by the local health authority (D13055-08, Préfecture des Bouches-du-Rhône, Marseille, France). The rats were deeply anesthetized with chloral hydrate (intraperitoneal, 200 mg kg^−1^) and killed by decapitation. The brain was rapidly extracted from the skull and put on an iced platform to isolate the hemispheres with a scalpel. A base was then created by making a cut in the frontal plane at ~2 mm from the posterior part of the cortex. The anterior part of the hemispheres was removed to obtain two blocks of 6–7 mm width that were glued with a cyanoacrylate glue on the slicing stage. Coronal cortical slices were cut with a VT1000S vibratome (Leica, Wetzlar, Germany) in a low-sodium, ice-cold solution containing (in mM) 280 sucrose, 26 NaHCO_3_, 10 D-glucose, 10 MgCl_2_, 1.3 KCl and 1 CaCl_2_, and they were bubbled with 95% O_2_/5% CO_2_, pH 7.4. Slices were recovered for ~1 h in a solution containing (in mM) 125 NaCl, 26 NaHCO_3_, 3 CaCl_2_, 2.5 KCl, 2 MgCl_2_, 0.8 NaH_2_PO_4_ and 10 D-glucose, and they were equilibrated with 95% O_2_/5% CO_2_.

Each cortical slice was transferred to a submerged slice chamber (Luigs & Neumann, Ratingen, Germany) mounted on an upright microscope (Olympus, Tokyo, Japan) equipped with a 40x water-immersion objective (Olympus, Tokyo, Japan). Pairs of L5 pyramidal neurons were visualized using DIC infrared video-microscopy.

Dual whole-cell recordings were obtained as detailed previously [51,52]. Nearby pyramidal neurons with axon initial segment and apical dendrites that run in parallel to the surface of the slice were selected for dual patch-clamp recordings. The external solution contained (in mM) 125 NaCl, 26 NaHCO_3_, 3 CaCl_2_, 2.5 KCl, 2 MgCl_2_, 0.8 NaH_2_PO_4_ and 10 D-glucose and was equilibrated with 95%0_2_/5% C0_2_. Patch pipettes (5–10 MΩ) pulled from borosilicate glass capillaries (World Precise Instruments, Sarasota, FL, USA) with a PC-100 micropipette puller (Narishige, Tokyo, Japan), were filled with a solution containing (in mM) 120 K-gluconate, 20 KCl, 0.5 EGTA, 10 HEPES, 2 Na_2_ATP, 0.3 NaGTP and 2 MgCl_2_, pH 7.4. Recordings were made at 34 °C in a temperature-controlled recording chamber (Luigs & Neumann, Ratingen, Germany). Classically, the presynaptic neuron was recorded in a current clamp with an Axoclamp 2B amplifier (Axon Instruments, Molecular Devices, San Jose, CA, USA) and the post-synaptic cell was recorded in a voltage clamp with an Axopatch 200B amplifier (Axon Instruments, Molecular Devices, San Jose, CA, USA). Pre- and post-synaptic cells were held at their resting membrane potential (~−60/−65 mV). The membrane potential was not corrected for the liquid junction potential (~−13 mV). Presynaptic APs were generated by injecting brief (5–10 ms) depolarizing pulses of current at a frequency of 0.3 Hz. All paired-pulse protocols were performed by triggering two presynaptic action potentials at a frequency of 20 Hz (i.e., an interval of 50 ms) in order to evoke two postsynaptic responses (EPSC1 and EPSC2). The paired-pulse ratio (PPR) was calculated as follows: PPR = (EPSC2/EPSC1) * 100. The voltage and current signals from the amplifiers were amplified (100X or 500X) and low-pass filtered (3 kHz), and the acquisition of 500 ms sequences was performed at 10–15 kHz with Acquis1 (G. Sadoc, CNRS, Gif-sur-Yvette, France) through an analogue–digital board (Digidata 1322A, Axon Instruments, Molecular Devices, San Jose, CA, USA).

Synaptic responses evoked by the stimulation of the presynaptic cell were averaged following an alignment of the presynaptic action potentials using automatic peak detection (Detectivent 4.0, N. Ankri, Unité de Neurobiologie des canaux Ioniques et de la Synapse (UNIS), Marseille, France) [26]. The presence or absence of a synaptic connection between two neurons was determined on the basis of averages over 30–50 individual traces, including synaptic failures, as previously described [51]. A monosynaptic connection was identified on the basis of 2 criteria: the latency between the presynaptic AP and the onset of the response in the postsynaptic neuron is short (i.e., ~1–5 ms) and stable with fluctuations below 1.5 ms. With this technique, even very small responses (<0.2 mV or <10 pA) could be easily detected in the post-synaptic recording. In practise, the smallest post-synaptic responses detected had an amplitude of 0.1 mV and 4 pA. Nevertheless, the analysis was restricted to a corpus of connections with a mean amplitude larger than 0.3 mV/10 pA. The latency of individual EPSCs was measured from the peak of the presynaptic AP measured in the cell body to 5% of the maximal EPSC amplitude [26,53].

d-ADF was tested by continuously depolarizing or hyperpolarizing the presynaptic membrane potential with an injection of holding current (±15–30 pA). Paired-pulse plasticity was tested using two depolarizing current steps to evoke two action potentials with a delay of 50 ms [54]. The coefficient of variation was analysed on individual traces [55]. Data are presented as means ± SEM, and a paired *t*-test performed with SigmaPlot (version 11, Softonic, Barcelona, Spain) was used for all comparisons.

## 3. Results

### 3.1. d-ADF at L5-L5 Synapses

**Figure 1:**
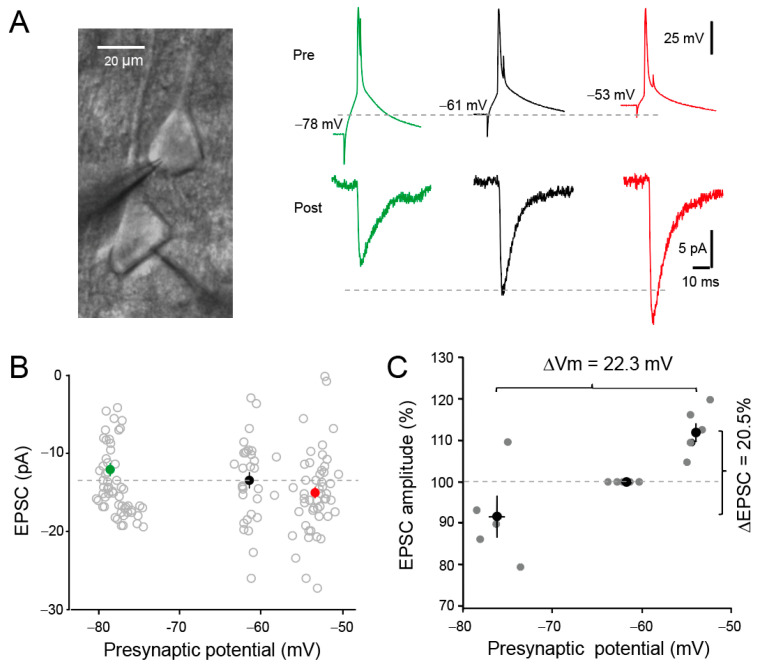
Induction of analogue-digital facilitation (d-ADF) in connected pairs of L5 pyramidal neurons. (**A**) Left, pairs of L5 pyramidal neurons during whole-cell recording. Right, depolarization-induced analogue-digital facilitation. Compared to the control situation (i.e., presynaptic membrane potential at rest: −61 mV, black traces), spike-evoked synaptic transmission is inhibited at hyperpolarized potential (green traces, −78 mV) but facilitated at depolarizing potential (red traces, −53 mV). (**B**) Analysis of the evoked synaptic responses as a function of presynaptic voltage shown in panel A. Gray circles correspond to individual data points and full circles to averages. Same-color symbols as in panel A. (**C**) Group data showing d-ADF. The variation in EPSC amplitude (ΔEPSC) amounts to 20.5% for a variation in presynaptic potential (ΔVm) of 22.3 mV, indicating that d-ADF amounts to ~1% per mV of presynaptic depolarization.

Pairs of connected L5 pyramidal neurons from the sensorimotor cortex of young rats were recorded in a whole-cell configuration. To induce depolarization-induced analogue–digital facilitation (d-ADF) and check the impact of the presynaptic membrane potential on the spike-evoked transmission, we changed the holding current from 0 to ±15–30 pA. Continuous presynaptic hyperpolarization from −61 to −78 mV reduced the amplitude of the spike-evoked EPSC, whereas continuous depolarization from −61 to −53 mV increased the EPSC amplitude (Figure 1A,B). On average, a shift in presynaptic membrane potential from −76.4 mV to −54.1 mV (i.e., corresponding to a ΔVm = 22.3 mV) induced an EPSC change from −91.4 to 111.9% (i.e., ΔEPSC = 20.5%; paired *t*-test, *p* < 0.05; Figure 1C), indicating that the facilitation index is ~1% per mV of presynaptic depolarization, as reported earlier [38,42]. We conclude that synaptic transmission is enhanced at synapses formed by pairs of L5-L5 neurons following slow changes in the membrane potential of the presynaptic neuron from hyperpolarization to depolarization.

### 3.2. d-ADF at L5-L5 Synapse Results from an Elevation in Pr

**Figure 2:**
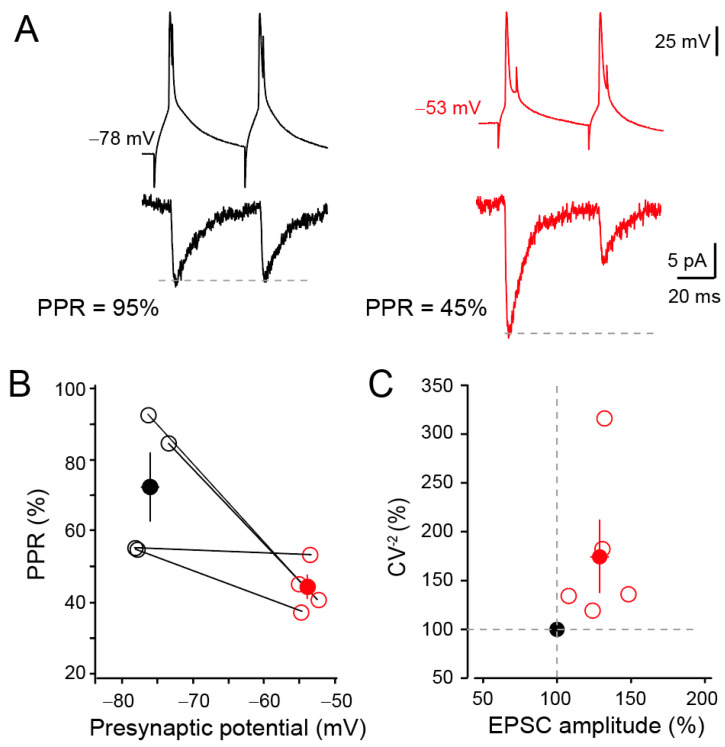
Presynaptic release of glutamate during d-ADF. (**A**) Paired-pulse ratio (PPR) is reduced during d-ADF. Left, when the presynaptic membrane potential is hyperpolarized (−78 mV), PPR is 95%. Right, at depolarized presynaptic potential (−53 mV), synaptic transmission is increased and PPR falls to 45%. (**B**) Group data showing the reduction in PPR in all the cell pairs tested. (**C**) Analysis of changes in CV^−2^ as a function of EPSC changes. Note the increase in CV^−2^ during EPSC enhancement (i.e., d-ADF).

In order to check the presynaptic origin of d-ADF, two successive presynaptic action potentials were triggered with a delay of 50 ms at hyperpolarized (mean: −76.7 ± 0.9 mV) and depolarized (mean: −53.8 ± 0.4 mV) membrane potentials. d-ADF was associated with a reduced paired-pulse ratio (PPR from 72 ± 10% in control to 44 ± 3% during d-ADF (Figure 2A,B)). To confirm the presynaptic origin of d-ADF, CV^−2^ (coefficient of variation at the power −2) of EPSC fluctuations was analysed. As expected, CV^−2^ was elevated (173 ± 37% of the control CV^−2^, *n* = 5; paired *t*-test *p* < 0.05) in parallel to that of EPSC amplitude (128 ± 7% of the control EPSC amplitude, *n* = 5, paired *t*-test, *p* < 0.05; Figure 2C), confirming an increase in *Pr* during d-ADF in L5 neurons, as reported earlier [38,42]. We conclude that *Pr* is elevated during d-ADF.

### 3.3. No Change in Synaptic Delay during d-ADF

**Figure 3:**
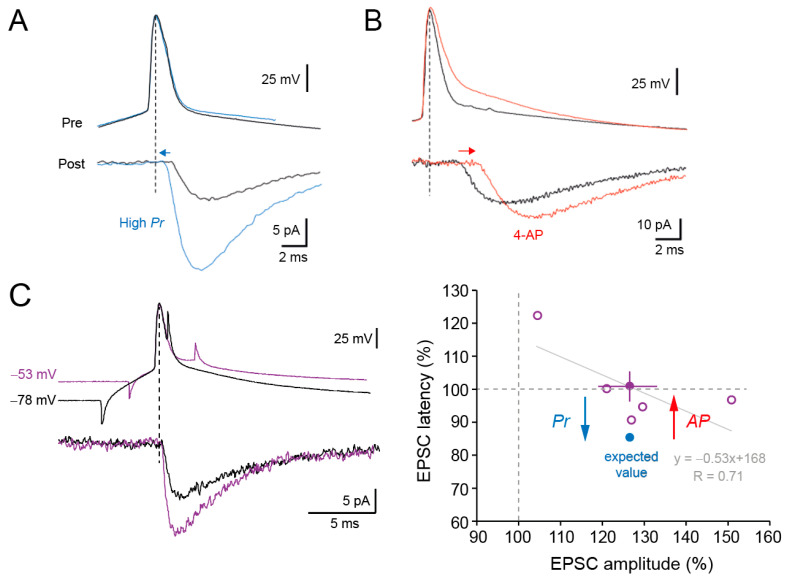
No change in synaptic delay during d-ADF. (**A**). A high release probability reduces the synaptic delay (taken from reference [26] with permission). The blue traces correspond to a high *Pr* whereas the black traces correspond to a low *Pr*. These data were recorded under spontaneous fluctuations in *Pr*. No change in the shape of the presynaptic AP is observed (see superimposed traces). (**B**). Action potential broadening induced by the application of 4-amino-pyridine (4-AP, red traces) delays synaptic latency (taken from reference [32] with permission). (**C**). Despite an elevation in *Pr* during d-ADF, EPSC latency is not changed. Left, representative traces at hyperpolarized presynaptic membrane potential (black traces) and at depolarized membrane potential (purple traces). Right, group data. Individual data correspond to open purple circles and the average to the filled purple circle. For an increase of +27% of EPSC amplitude, EPSC latency should be reduced by ~15% (expected value, blue disc). Yet, no change is observed on average (purple disc). The increase in synaptic delay induced by spike broadening due to the voltage inactivation of Kv1 channels is thought to compensate for the reduction in synaptic delay induced by elevated *Pr*. Grey line, linear regression of the data points with the corresponding equation.

We next determined whether synaptic delay was modified during d-ADF. Synaptic delay is reduced (Figure 3A) when the release probability is elevated [26] and augmented (Figure 3B) when the duration of the presynaptic AP is broadened by the pharmacological blockade of Kv1 channels [32]. For a synaptic facilitation of ~25% (see Figure 2C), a reduction in latency by ~15% is observed (Figure 3C) according to our previous findings [26]. Whereas *Pr* is clearly elevated during d-ADF, no change in synaptic delay is observed (101 ± 6% of the control delay, *n* = 5, paired *t*-test, *p* > 0.5; Figure 3C).

In cortical and hippocampal pyramidal neurons, d-ADF is due to the voltage inactivation of presynaptic Kv1 channels that broadens the presynaptic AP [38,42]. Thus, the absence of change in synaptic delay can be explained by the superposition of the two processes, i.e., the reduction in synaptic delay induced by the elevation of *Pr* is compensated for by the increase in synaptic delay produced by the depolarization-induced AP broadening (Figure 3C). Supporting this hypothesis, changes in synaptic delay were found to be anticorrelated with the magnitude of d-ADF (linear regression, R = 0.71; Figure 3C), although, on average, no change in synaptic delay is observed for the whole population of L5-L5 synapses. In fact, the larger the facilitation, the shorter the delay latency. We conclude that d-ADF is, on average, not associated with any changes in synaptic delay.

## 4. Discussion

The synaptic delay at cortical L5-L5 synapse is determined by both the release probability (*Pr*) and the presynaptic AP width in opposite ways. An enhanced *Pr* reduces synaptic delay [26], whereas presynaptic AP broadening prolongs synaptic delay [32]. While *Pr* is clearly enhanced during d-ADF (as indicated by the reduction in PPR and the increase in CV^−2^), we show that the synaptic delay is surprisingly unchanged. Indeed, for a presynaptic increase in synaptic transmission of ~25%, the synaptic delay should be reduced by ~15% [26]. The simplest explanation for this observation is that opposite changes in synaptic delay occur during d-ADF, i.e., the *Pr*-dependent reduction in synaptic delay is compensated for by the AP-dependent increase in synaptic delay. This assumption is further supported by the fact that changes in synaptic delay were anticorrelated with the magnitude of d-ADF. A small d-ADF corresponds to a modest increase in *Pr*, and so to a small reduction in synaptic delay, whereas a large d-ADF corresponds to a large increase in *Pr*, and so to a large reduction in synaptic delay. Thus, in contrast to classical short-term presynaptic plasticity (PPF and PPD) that only results from changes in *Pr* and displays clear modulation in synaptic delay [26,56], no changes in synaptic delay are observed during d-ADF.

A similar compensatory phenomenon has already been reported at the synapse formed by the mossy-fibre with the CA3 pyramidal cell during repetitive stimulation (25 times) of the presynaptic input [32]. In this study, the synaptic delay was found to be reduced by ~1 ms following the first three stimuli, but it progressively increased by the fifth stimulus because of the cumulative inactivation of presynaptic Kv1 channels [57]. In fact, no increase in synaptic delay was observed in the presence of the Kv1 channel blocker. Other context-dependent forms of synaptic facilitation such as hyperpolarization-induced ADF (h-ADF, [39]) or input-synchrony-dependent facilitation (ISF, [40]) are due to the modulation of presynaptic AP amplitude as a result of changes in the inactivation of axonal Nav channels. As the reduction of presynaptic AP shortens synaptic delay [32], it is conceivable that no changes in synaptic delay would also be observed during both h-ADF [39] and ISF [40], as the reduced delay due to the elevation of *Pr* would be, again, compensated for by an increased delay resulting from the increased AP amplitude.

Synaptic delay is inversely proportional to the presynaptic calcium concentration [24]. Calcium concentration is supposed to be slightly elevated during d-ADF for the more proximal synapses (i.e., up to ~150–200 µm from the presynaptic cell body) as a result of the activation of P/Q-type calcium channels [38]. However, this mechanism cannot account for the stability of the synaptic delay observed during d-ADF as it should reinforce the reduction in synaptic delay.

Another possible explanation to account for the stability of the synaptic delay during d-ADF would result from the inactivation of voltage-gated channels by the depolarization imposed to the presynaptic cell. Nav channels are critical for AP conduction along the axon, and any reduction in the sodium current such as that produced by the voltage-inactivation of Nav channels induced by constant depolarization may reduce conduction speeds. However, the fact that synaptic delay changes were anticorrelated with EPSP changes cannot be explained by the voltage-inactivation of Nav channels. Furthermore, the inactivation of Nav channels would reduce the amplitude of the presynaptic AP. The application of a low concentration of tetrodotoxin (TTX, 40 nM) similarly reduces the amplitude of the presynaptic AP and reduces the synaptic delay [32]. But here, again, this would not counteract the reduction in synaptic delay produced by an elevated *Pr*. 

The conduction time along the axon depends on several parameters such as the axon diameter [58] and the membrane potential of oligodendrocytes. An increase in axon diameter observed following a high-frequency stimulation shortens conduction time [59]. The depolarization of oligodendrocytes also reduces the conduction time [60]. However, these mechanisms are unlikely to occur during d-ADF as the rate of the stimulation was kept constant in our experiments, and the change in membrane potential was only imposed in the presynaptic neuron. However, the transmission of the change in membrane potential imposed in the neuron to nearby astrocytes through ephaptic contacts cannot be totally excluded [61].

In this study, presynaptic APs recorded in the soma serve as temporal references for measuring synaptic delay. This reference is correct if conduction time remains constant. However, conduction time may vary during d-ADF as the depolarization of the axon during d-ADF would increase conduction velocity because the AP threshold would be easier to reach [62,63]. Nevertheless, this mechanism cannot account for the lack of change in synaptic delay observed during d-ADF as it would further reduce the synaptic delay in addition to the *Pr*-dependent mechanism. In conclusion, the lack of change in synaptic delay during d-ADF at the cortical synapse is probably due to the compensation for *Pr*-dependent and AP-broadening-dependent changes in synaptic delay. 

The functional implications of synaptic modulation without changes in synaptic delay are rather difficult to precisely evaluate. However, d-ADF is supposed to occur during “down” to “up” state transitions [41,64] or, more generally, during global shifts in membrane potential that occur during sleep in the mammalian brain [65,66]. “Up” states generally last 0.5 to 2 s. While the voltage transitions were much longer in our experiments, we believe that the conclusions made here remain valid during “up” and “down” transitions. In fact, the voltage inactivation Kv1 channels by 0.5–2 s are similar to those produced by constant depolarization as the time constant of Kv1 channel inactivation is ~1 s. If changes in synaptic delay represent a neural code [11,26], it could be important that synaptic timing remains constant during synaptic facilitation that may occur during slow-wave sleep and memory consolidation [67,68]. Further investigations will be necessary to understand the precise role of synaptic timing in brain function and coding.

## 5. Conclusions

We report here that synaptic delay remains constant during the analogue–digital facilitation of synaptic transmissions induced at L5-L5 neocortical synapses by slow changes in presynaptic membrane potential from hyperpolarized to depolarized levels. This form of synaptic facilitation is due to an enhancement of the presynaptic release probability caused by the broadening of the action potential in the presynaptic terminal. It has been previously shown that the enhancement of presynaptic release reduces synaptic delay while the action potential broadening due to the voltage inactivation of potassium channels increases this delay. While no changes in synaptic delay were observed on average, changes in synaptic delay were found to be anticorrelated with the magnitude of d-ADF. Overall, our results indicate that the two modulations in synaptic delay produced by *Pr* and AP broadening cancel each other to maintain an unchanged synaptic timing during the analogue–digital facilitation induced by depolarization.

## Data Availability

All data reported in this study are included in the manuscript.
